# Facial Expressions, Emotions, and Sign Languages

**DOI:** 10.3389/fpsyg.2013.00115

**Published:** 2013-03-11

**Authors:** Eeva A. Elliott, Arthur M. Jacobs

**Affiliations:** ^1^Department of Experimental and Neurocognitive Psychology, Freie Universität BerlinBerlin, Germany; ^2^Dahlem Institute for Neuroimaging of EmotionBerlin, Germany

**Keywords:** sign language, facial expression, emotion

## Abstract

Facial expressions are used by humans to convey various types of meaning in various contexts. The range of meanings spans basic possibly innate socio-emotional concepts such as “surprise” to complex and culture specific concepts such as “carelessly.” The range of contexts in which humans use facial expressions spans responses to events in the environment to particular linguistic constructions within sign languages. In this mini review we summarize findings on the use and acquisition of facial expressions by signers and present a unified account of the range of facial expressions used by referring to three dimensions on which facial expressions vary: semantic, compositional, and iconic.

## Introduction

Humans perceive facial expressions as conveying meaning, but where do they come from and what exactly do they mean? Based on observations of facial expressions typically associated with emotions Darwin (1904) hypothesized that they must have had some instrumental purpose in evolutionary history. For example, lifting the eyebrows might have helped our ancestors respond to unexpected environmental events by widening the visual field and therefore enabling them to see more. Even though their instrumental function may have been lost, the facial expression remains in humans as part of our biological endowment and therefore we still lift our eyebrows when something surprising happens in the environment whether seeing more is of any value or not. Following this tradition Ekman (1979, 1992) claimed that there is a set of facial expressions that are innate, and they mean that the person making that face is experiencing an emotion; i.e., brow raising means “I feel surprised.” He also claimed that there are culturally acquired facial expressions used to modulate the innate emotional expressions, so-called display rules, and also others that are used for communication. Examples of the latter type are; (a) an eyebrow flash used to mean “hello,” (b) eyebrow movements during speech that emphasize certain words. According to this view, some facial expressions are “read outs” of inner emotional states and the fact that they have a meaning to the observer is incidental, while others are used specifically for communication and are thus in some sense intentionally meaningful.

However, Fridlund (1997) claimed that there are no “read outs” of inner emotional states; rather, what are usually regarded as emotional expressions evolved to communicate intentions. That is, raised eyebrows do not mean “I am surprised,” but might mean “Something happened; I am going to find out what.” From this view all facial expressions evolved for communicative purposes.

The past 30 years of linguistic research on sign languages have revealed that there are facial expressions which are used together with manual signs and function as phonological features, morphemes, and syntactic/prosodic markers, for example brow raising marking conditional clauses (Liddell, 1980; Dachkovsky and Sandler, 2009). These facial expressions are clearly communicative in nature and they are used in combination with other meaningful movements (those of the hands).

In sum, there is evidence that facial expressions mean things ranging from possibly universal messages, i.e., “I am surprised”/“Something happened!” to culture specific learned meanings; i.e., “hello,” to culture specific meanings that can take part in larger composite structures with other meaningful elements, i.e., the conditional clause marker in sign languages.

How does one account for the range of meanings and uses of facial expressions? Following Wierzbicka (1999), we argue that facial expressions are semiotic units (form-meaning pairings) that can be analyzed with the same semantic methodology used to analyze words (see, Wierzbicka, 1996, for an account of her methodology). Two further working assumptions that we adopt from Wierzbicka (1999, p. 185) are: (a) some facial configurations have identifiable context independent meanings; (b) some facial expressions have a universal meaning which can be interpreted without reference to culture. Assumption (a) is also made by Dachkovsky and Sandler (2009), although as far as we understand, they limit this claim to facial expressions used as prosodic units. Assumption (b) is shared by Ekman. Note that in general a strong argument can be made that some facial expressions are innate because they are also produced by congenitally blind persons (Matsumoto and Willingham, 2009), but determining their meaning is a matter of greater controversy.

To illustrate the controversy, we will briefly discuss the meaning of brow raise, as we use this facial expression as an example throughout this paper. Ekman (1992) proposes that it means “I am surprised,” but we adopt Wierzbicka’s (1999, p. 205) suggestion that it means “I want to know more (about this).” We adopt Wierzbicka’s interpretation for the following reasons: Wierzbicka points out that the term “surprise” is not universal, it is part of Anglo language and culture. She suggests instead that the meanings of facial expressions can be better expressed using terms from the natural semantic metalanguage (Wierzbicka, 1996) for which she has some evidence of universality. Furthermore, it seems to us that part of the meaning of being surprised is, in fact “wanting to know more about this [unexpected event that just occurred],” so the two interpretations are not completely incompatible. However we find Wierzbicka’s definition more general with the power to cover the use of brow raise in relation to emotion and in sign languages, so it is this one that we adopt, acknowledging that currently there is no consensus on the matter.

As regards facial expressions in general, we propose that their differences and similarities can be explained in terms of three dimensions: semantic, iconic and compositional. These dimensions are derived from our first working assumption; that some facial expressions are semiotic units (form-meaning pairings). The semantic dimension refers to the meaning part of the semiotic unit, the iconicity dimension to the nature of the relationship between the form and the meaning, and compositionality to the way the semiotic unit can combine with other semiotic units to form complex semiotic structures. The semantic dimension spans meanings that are universal to those which are culture specific. The iconic dimension spans the varying degrees in which facial expressions resemble their meaning. The compositionality dimension spans the degrees in which facial expressions readily combine with other semiotic units to form complex structures. A similar proposition to this has been made to account for the range of hand movements used by humans, covering the co-speech gestures of hearing individuals to signing by Deaf individuals (McNeill, 1992). In this mini review we summarize evidence from acquisition of facial expressions by signers to support our view. We first present a brief overview of the role of the face in sign language structure. We then describe the proposed dimensions and the findings on acquisition of facial expressions by Deaf signers that support them, after which we come to a conclusion. Note that to the best of our knowledge currently there only exists acquisitional data on non-manuals for American Sign Language (ASL) and so the examples below all refer to ASL.

## Sign Languages and The Role of the Face

Sign languages are the naturally occurring linguistic systems that arise within a Deaf community and, like spoken languages, have phonological, lexical, and syntactic levels of structure (e.g., Liddell, 2003; Sandler and Lillo-Martin, 2006). Cognitive and neurocognitive data provide evidence that signed and spoken languages are processed in a similar manner; for example, they show similar lexical access effects (Baus et al., 2008; Carreiras et al., 2008) and they are supported by similar brain regions (Campbell et al., 2008).

Facial and head movements are used in sign languages at all levels of linguistic structure. At the phonological level some signs have an obligatory facial component in their citation form (Liddell, 1980; Woll, 2001). There are facial morphemes in ASL such as the adverbial “th” meaning “carelessly” (McIntire and Reilly, 1988; Crasborn et al., 2008). Facial actions mark relative clauses, content questions and conditionals, amongst others, although there is some controversy whether these markings should be regarded as syntactic or prosodic (cf. Liddell, 1980; Baker-Shenk, 1983; Aarons et al., 1992; Nespor and Sandler, 1999; Sandler, 1999; Wilbur and Patschke, 1999; Neidle et al., 2000; Dachkovsky and Sandler, 2009; Wilbur, 2009). Signers also use the face to gesture (Sandler, 2009). Below we describe how these uses of the face can be described in terms of three dimensions; semantic, compositional, and iconic with evidence from facial expression acquisition.

## The Semantic Dimension

The semantic dimension refers to the meaning part of a semiotic unit. It has been proposed, especially for the meanings of facial expressions, that there are universal meanings and culture specific meanings. Eyebrow raise is considered a unit with a universal meaning, and we adopt the suggestion that it means “I want to know more (about this).”

The brow raise appears to be used both with and without accompanying speech. Context can give it additional meaning beyond “I want to know more (about this),” however we argue that even when more meaning is added by context it always retains its universal meaning. For example, hearing people may use brow raise while asking a yes-no question (Ekman, 1979), and when they are confronted with something unexpected in the environment. In both cases it still retains the meaning “I want to know more (about this)” but in the former case it is related to the words in the question and in the latter to the event. Within sign languages too, brow raise is used in different contexts; it can mark yes-no questions and the antecedent of conditionals. Dachkovsky and Sandler (2009, p. 300) propose that despite these different linguistic contexts eyebrow raise has one meaning, namely “[…] the intermediate or intonational phrase marked by [brow raise] is to be followed by another phrase, produced either by the interlocutor or another.” We find Dachkovsky and Sandler’s interpretation compatible with that of “I want to know more (about this)” or a similar formulation such as “more information is coming.”

On the culture specific end of the semantic dimension lays, for example, the ASL adverbial “th” (carelessly), conveyed by sticking one’s tongue out slightly between closed lips and tilting the head. In saying that “carelessly” is a culture specific concept, we mean that not all languages have labeled the complex set of behaviors and attitudes that make up the meaning of “carelessly” with a word/sign. We do not mean, however, that the concept cannot be explained to someone whose language does not have a word for it.

The semantically universal facial expressions are logically the first to appear in acquisition. By 0:2 children are raising their brows in what Izard et al. (1995) call an expression of “interest,” but which we refer to as “I want to know more (about this).” Culture specific facial expressions such as the negating headshake appear at approximately 1:0 but they are not yet combined with signing (Anderson and Reilly, 1997; Reilly, 2005).

## The Iconicity Dimension

We use the term iconicity to mean a form-meaning resemblance. Resemblance by its nature is a matter of degree. Some facial expressions resemble their meanings to a greater degree than others. The relation between the form brow raise and the meaning “I want to know more (about this)” is iconic since raising one’s brows to see more is a metaphorical icon (Taub, 2001) of wanting to know more. The adverbial “th” (carelessly) also seems to be iconic since the slight tongue protrusion and head tilt could resemble the face of a person behaving carelessly. We do not have data on facial expressions used either by hearing or deaf people that are completely arbitrarily related to their meaning; however we think this is in principle possible because many semiotic units, especially in spoken language, do not appear to display any form-meaning resemblance. We propose the eye-wink, used in some Anglo cultures to mean “I am not serious,” as an example of a facial action arbitrarily related to a meaning.

In acquisition, since the universal expressions appear first, and since universal meanings would seem to necessarily have a form that is motivated by meaning (Wierzbicka, 1999, p. 213), therefore iconicity precedes arbitrariness. Even when signing children start combining expressions with signs at (1:6), the first types they use are emotion related facial expressions with emotion concept signs (McIntire and Reilly, 1988; Reilly et al., 1990b).

## The Compositionality Dimension

Above we saw that brow raise can be used alone or in combination with other semiotic units such as words, i.e., it is compositional. In sign languages brow raise can be used together with manual signs (which are equivalent to spoken words). In spoken languages brow raise can also be used together with words however within sign languages there seem to be more restrictions on how brow raise is combined with signs/words. The first major difference is that in some sign languages brow raise is obligatory in yes-no questions (Dachkovsky and Sandler, 2009), while in spoken languages it is not. The second difference is that facial expressions that take part in composite sign structures seem to be more strictly timed to the onset and offset of signs/words compared to spoken languages (Veinberg and Wilbur, 1990). It would seem that there is an increase in the combinatorial options for facial expressions when shifting from use of the face with spoken language to use of the face as part of signing similar to that proposed for gesticulation and sign language in McNeill (1992).

Not all facial expressions have to appear in composite structures; however we are not aware of the existence of a facial expression that disallows combination in all cases. For example it seems that even emblematic facial expressions that usually stand alone, such as the “hello” eyebrow flash mentioned above, could be used to replace words in a sentence. However, our point is that some facial expressions are more readily combined with other semiotic units than others, and that there are degrees in the regularity of composite structures, i.e., the combination of brow raise with words in sign languages is more regular in occurrence and timing than in spoken languages.

The acquisition of full mastery of the combinatorial conventions of facial expressions in sign language appears to last at least 7 years. The first combination of facial expressions with other semiotic units by signers happens at about 1:6. These facial actions appear to be phonological features. This is also when a manual sign for negation appears, but the child does not combine it with their headshake until 2 months later (1:8). At 2 years of age the first facial adverbials appear. At 2:5 children use facial expressions to depict other people’s emotions and at 3:0 use the break in eye contact and mimicry of others to mark role-shift. 3:0 is also the age when children use the manual sign for “if” but only at 5:0 do they start to use it with brow raise and only at 7:0 are they fully approximating adult production of conditionals (Reilly et al., 1990a; Reilly, 2005).

## Conclusion

In terms of the three proposed dimensions, as children acquire facial expressions they move from innate universal concepts mapped onto iconic forms produced in holistic structures to culture specific concepts, conventional form-meaning mappings, and increasingly complex composite structures. More data on facial expression acquisition in sign languages other than ASL, as well as data on the development and use of facial expressions in spoken language, will help to clarify what concepts and forms are universal (if any).

We find it important to note that our continua do not explain *how* children acquire facial expressions, rather they make a strong claim regarding *what* it is that children acquire: semiotic units and the knowledge of how to combine them into more complex semiotic units. This perspective contrasts with views claiming that emotion related facial expressions, facial expressions used by hearing people during conversation, and facial expressions used by signers while signing should be treated as distinct phenomena. We find it important to first accurately characterize the “what” of facial expression acquisition as this necessarily constrains possible answers as to how humans acquire facial expressions.

## Conflict of Interest Statement

The authors declare that the research was conducted in the absence of any commercial or financial relationships that could be construed as a potential conflict of interest.
